# Supplementary material for: Evaluation study of effect of virtual care education on healthcare providers’ knowledge, confidence, and satisfaction
Source: PeerJ. 2025 Nov 20;13:e20414. doi: 10.7717/peerj.20414 (PMC12640642; doi:10.7717/peerj.20414)
Supplement: Supplemental Information 1 — Surveys administered at pre-, mid-, and post-module time points assessing knowledge, confidence, and satisfaction in virtual care delivery. [file peerj-13-20414-s001.docx]

**Appendix A: Surveys**

Survey 1

*Instructions: Please read the following questions and select the most appropriate response for each one. At the end of the quiz, click the Submit button to complete the Pre-Test.*

1. Which of the following options best describes the benefits and key considerations of conducting virtual care appointments?

○ Increased convenience and flexibility for both patients and healthcare providers.

○ Improved accuracy of diagnosis and treatment outcomes compared to in-person visits.

○ Limited accessibility, excluding patients without access to technology or stable internet connection.

○ Heightened risk of infectious disease transmission due to close physical proximity.

○ Enhanced patient engagement and empowerment in managing their own healthcare.

2. Which of the following options best identifies the technological requirements and setup required to conduct optimal virtual care?

○ High-speed internet connection, secure communication platforms, and compatible devices.

○ Advanced medical equipment and specialized software for remote diagnostics.

○ Physical presence of a healthcare professional at the patient's location.

○ Virtual reality headsets and augmented reality devices for immersive virtual care experiences.

3. Which of the following options best recognizes how to integrate virtual care delivery into existing practice workflows?

○ Continuing with the same workflows and processes without any modifications.

○ Designating a separate team solely responsible for virtual care delivery.

○ Assessing current workflows and adapting them to incorporate virtual care components.

○ Implementing virtual care as a standalone service independent of existing workflows.

4. Which of the following options best describes the clinical skills required to deliver optimal virtual care encounters?

○ Proficiency in performing physical examinations.

○ Strong knowledge of medical billing and coding.

○ Effective communication and active listening skills.

○ Expertise in surgical procedures.

5. Which of the following options best describes how to prepare patients for virtual care sessions?

○ Providing detailed instructions on how to perform a physical examination on themselves.

○ Recommending patients to consult multiple healthcare providers simultaneously.

○ Ensuring patients have access to a stable internet connection and compatible devices.

○ Encouraging patients to delay seeking medical advice until an in-person visit is possible.

6. Which of the following options is a regulatory consideration for providing virtual care in Newfoundland and Labrador?

○ No specific regulations or legal considerations exist for virtual care in Newfoundland and Labrador.

○ Compliance with privacy and security regulations for patient data protection is mandatory.

○ Full licensure is required for healthcare providers delivering virtual care services.

○ Virtual care is exempt from liability and malpractice claims.

*Instructions: Select your level of confidence.*

*
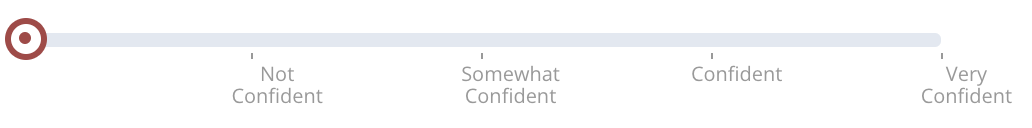
*

1. I can describe the benefits and key considerations of conducting virtual care appointments.

2. I can identify the technological requirements and setup required to conduct optimal virtual care.

3. I recognize how to integrate virtual care delivery into my existing practice workflows.

4. I can discuss the clinical implications for delivering optimal virtual care encounters.

5. I can explain how to prepare patients for virtual care sessions.

6. I can summarize the key regulatory and legal considerations in providing virtual care in Newfoundland and Labrador.

Survey 2

*Case Scenario: Dr. Smith is a family physician running a busy primary care practice in a suburban area. Due to the increasing demand for virtual care and the need to adapt to changing patient expectations, Dr. Smith decides to integrate virtual care into their practice.*

1. Assessing the Practice: What considerations should Dr. Smith examine in order to determine the readiness of their practice for virtual care?

2. Preparing the Practice: Assuming that Dr. Smith decides to pursue the adoption of virtual care delivery in their practice, what other things should they plan for in order to prepare?

3. Establishing the Workflow: Dr. Smith recognizes the importance of managing the workflow in their clinic to effectively integrate virtual care services. How might Dr. Smith approach understanding and modifying existing workflows in their clinic?

4. Preparing Patients for Virtual Care: What strategies might Dr. Smith use to help prepare their patients?

5. The First Patient: How would Dr. Smith determine if this patient is a good candidate for virtual care?

6. Preparing for the First Visit: What things should Dr. Smith consider in terms of setting up the physical clinical space for the virtual care visit?

7. Conducting the First Visit: What things should Dr. Smith attend to during his virtual care session with Mr. Anderson?

8. Key Learning Points: After completing this module on virtual care, what are some of the key learning points that you are taking away from this experience?

Survey 3

*Please read the following questions and select the most appropriate response for each one. At the end of the quiz, click the Submit button to complete the Post-Test. You will be presented with feedback on which questions you answered correctly, which questions you answered incorrectly, and a brief rationale supporting the correct answer for each question.*

1. Which of the following options best describes the benefits and key considerations of conducting virtual care appointments?

○ Increased convenience and flexibility for both patients and healthcare providers.

○ Improved accuracy of diagnosis and treatment outcomes compared to in-person visits.

○ Limited accessibility, excluding patients without access to technology or stable internet connection.

○ Heightened risk of infectious disease transmission due to close physical proximity.

○ Enhanced patient engagement and empowerment in managing their own healthcare.

2. Which of the following options best identifies the technological requirements and setup required to conduct optimal virtual care?

○ High-speed internet connection, secure communication platforms, and compatible devices.

○ Advanced medical equipment and specialized software for remote diagnostics.

○ Physical presence of a healthcare professional at the patient's location.

○ Virtual reality headsets and augmented reality devices for immersive virtual care experiences.

3. Which of the following options best recognizes how to integrate virtual care delivery into existing practice workflows?

○ Continuing with the same workflows and processes without any modifications.

○ Designating a separate team solely responsible for virtual care delivery.

○ Assessing current workflows and adapting them to incorporate virtual care components.

○ Implementing virtual care as a standalone service independent of existing workflows.

4. Which of the following options best describes the clinical skills required to deliver optimal virtual care encounters?

○ Proficiency in performing physical examinations.

○ Strong knowledge of medical billing and coding.

○ Effective communication and active listening skills.

○ Expertise in surgical procedures.

5. Which of the following options best describes how to prepare patients for virtual care sessions?

○ Providing detailed instructions on how to perform a physical examination on themselves.

○ Recommending patients to consult multiple healthcare providers simultaneously.

○ Ensuring patients have access to a stable internet connection and compatible devices.

○ Encouraging patients to delay seeking medical advice until an in-person visit is possible.

6. Which of the following options is a regulatory consideration for providing virtual care in Newfoundland and Labrador?

○ No specific regulations or legal considerations exist for virtual care in Newfoundland and Labrador.

○ Compliance with privacy and security regulations for patient data protection is mandatory.

○ Full licensure is required for healthcare providers delivering virtual care services.

○ Virtual care is exempt from liability and malpractice claims.

*Instructions: Select your level of confidence.*

*
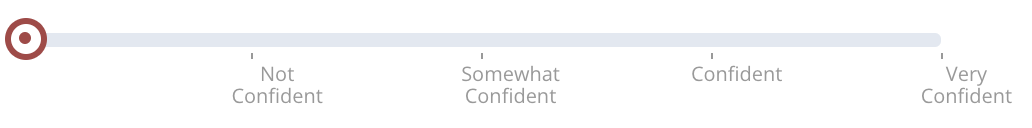
*

1. I can describe the benefits and key considerations of conducting virtual care appointments.

2. I can identify the technological requirements and setup required to conduct optimal virtual care.

3. I recognize how to integrate virtual care delivery into my existing practice workflows.

4. I can discuss the clinical implications for delivering optimal virtual care encounters.

5. I can explain how to prepare patients for virtual care sessions.

6. I can summarize the key regulatory and legal considerations in providing virtual care in Newfoundland and Labrador.

Survey 4

Course Content:


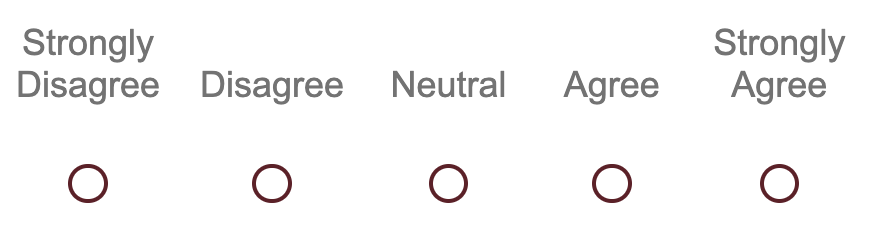


1. Addressed my learning needs.

2. Enhanced my knowledge.

Course Navigation:


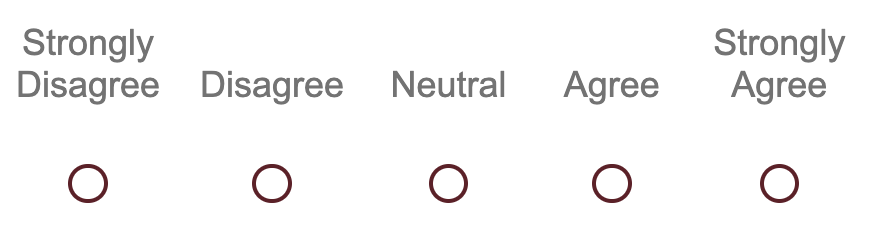


1. Instruction on use of and access to the course was helpful.

2. The pages were well-organized.

3. The pages were easy to navigate.

4. I received adequate help with technical problems.

Interactive Components:


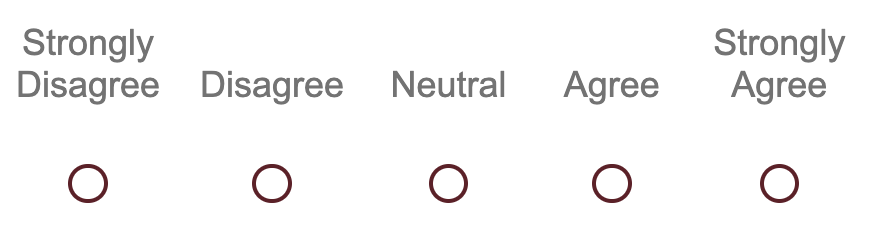


1. Participation in the discussion activities enhanced my understanding of the content.

2. Being provided with the opportunity to communicate with peers was helpful.

3. The discussion component was easy to use.

4. The “Ask the Expert” option addressed my learning needs.

1. What did you like about this course?

2. How could we have improved this course?
