# Supplementary material for: Evaluation study of effect of virtual care education on healthcare providers’ knowledge, confidence, and satisfaction
Source: PeerJ. 2025 Nov 20;13:e20414. doi: 10.7717/peerj.20414 (PMC12640642; doi:10.7717/peerj.20414)
Supplement: Supplemental Information 2 [file peerj-13-20414-s002.docx]

# Pre-Test

*Please read the following questions and select the most appropriate response for each one. At the end of the assessment, click the Submit button to complete the Pre-Test.*

**Content Questions**

Q1. Which of the following options best describes the benefits and key considerations of conducting virtual care appointments?

1. Increased convenience and flexibility for both patients and healthcare providers.
2. Improved accuracy of diagnosis and treatment outcomes compared to in-person visits.
3. Limited accessibility, excluding patients without access to technology or stable internet connection.
4. Heightened risk of infectious disease transmission due to close physical proximity.
5. Enhanced patient engagement and empowerment in managing their own healthcare.

**Feedback:**

Correct answer: A. Increased convenience and flexibility for both patients and healthcare providers.

Connolly SL, Gifford AL, Miller CJ, Bauer MS, Lehmann LS, Charness ME. Provider Perceptions of Virtual Care During the Coronavirus Disease 2019 Pandemic: A Multispecialty Survey Study. Med Care. 2021 Jul 1;59(7):646-652. doi: 10.1097/MLR.0000000000001562. PMID: 34009880; PMCID: PMC8191369.

Q2. Which of the following options best identifies the technological requirements and setup required to conduct optimal virtual care?

1. High-speed internet connection, secure communication platforms, and compatible devices.
2. Advanced medical equipment and specialized software for remote diagnostics.
3. Physical presence of a healthcare professional at the patient's location.
4. Virtual reality headsets and augmented reality devices for immersive virtual care experiences.

**Feedback:**

Correct answer: A. High-speed internet connection, secure communication platforms, and compatible devices.

Bokolo A. Use of Telemedicine and Virtual Care for Remote Treatment in Response to COVID-19 Pandemic. J Med Syst. 2020: 44(132). <https://doi.org/10.1007/s10916-020-01596-5>

Q3. Which of the following options best recognizes how to integrate virtual care delivery into existing practice workflows?

1. Continuing with the same workflows and processes without any modifications.
2. Designating a separate team solely responsible for virtual care delivery.
3. Assessing current workflows and adapting them to incorporate virtual care components.
4. Implementing virtual care as a standalone service independent of existing workflows.

**Feedback:**

Correct answer: C. Assessing current workflows and adapting them to incorporate virtual care components.

Hodgkins, M., Barron, M., Jevaji, S. et al. Physician requirements for adoption of telehealth following the SARS-CoV-2 pandemic. npj Digit. Med. 4, 19 (2021). https://doi.org/10.1038/s41746-021-00390-y

Q4. Which of the following options best describes the clinical skills required to deliver optimal virtual care encounters?

1. Proficiency in performing physical examinations.
2. Strong knowledge of medical billing and coding.
3. Effective communication and active listening skills.
4. Expertise in surgical procedures.

**Feedback:**

Correct answer: C. Effective communication and active listening skills.

Galpin K, Sikka N, King SL, Horvath KA, Shipman SA, AAMC Telehealth Advisory Committee. Expert consensus: Telehealth skills for health care professionals. Telemedicine and e-Health. 2021 Jul 1;27(7):820-4.

Q5. Which of the following options best describes how to prepare patients for virtual care sessions?

1. Providing detailed instructions on how to perform a physical examination on themselves.
2. Recommending patients to consult multiple healthcare providers simultaneously.
3. Ensuring patients have access to a stable internet connection and compatible devices.
4. Encouraging patients to delay seeking medical advice until an in-person visit is possible.

**Feedback:**

Correct answer: C. Ensuring patients have access to a stable internet connection and compatible devices.

Knierim K, Palmer C, Kramer ES, Rodriguez RS, VanWyk J, Shmerling A, Smith P, Holmstrom H, Bacak BS, Levey SM, Staton EW. Lessons learned during COVID-19 that can move telehealth in primary care forward. The Journal of the American Board of Family Medicine. 2021 Feb 1;34(Supplement):S196-202.

Q6. Which of the following options is a regulatory consideration for providing virtual care in Newfoundland and Labrador?

1. No specific regulations or legal considerations exist for virtual care in Newfoundland and Labrador.
2. Compliance with privacy and security regulations for patient data protection is mandatory.
3. All virtual care platforms must have a seal of approval from the Newfoundland and Labrador Tourism Board.
4. Virtual care is exempt from liability and malpractice claims.

**Feedback:**

Correct answer: B Compliance with privacy and security regulations for patient data protection is mandatory.

[College of Physicians and Surgeons of Newfoundland and Labrador. Standard of Practice: Virtual Care (2021).](https://cpsnl.ca/wp-content/uploads/2022/09/Virtual-Care-2021.pdf)

**Confidence Questions:**

Q7. I can describe the benefits and key considerations of conducting virtual care appointments.

| Not Confident | Somewhat Confident | Confident | Very Confident |
| --- | --- | --- | --- |
| 1 | 2 | 3 | 4 |

Q8. I can identify the technological requirements and setup required to conduct optimal virtual care.

| Not Confident | Somewhat Confident | Confident | Very Confident |
| --- | --- | --- | --- |
| 1 | 2 | 3 | 4 |

Q9. I recognize how to integrate virtual care delivery into my existing practice workflows.

| Not Confident | Somewhat Confident | Confident | Very Confident |
| --- | --- | --- | --- |
| 1 | 2 | 3 | 4 |

Q10. I can discuss the clinical implications for delivering optimal virtual care encounters.

| Not Confident | Somewhat Confident | Confident | Very Confident |
| --- | --- | --- | --- |
| 1 | 2 | 3 | 4 |

Q11. I can explain how to prepare patients for virtual care sessions.

| Not Confident | Somewhat Confident | Confident | Very Confident |
| --- | --- | --- | --- |
| 1 | 2 | 3 | 4 |

Q12. I can summarize the key regulatory and legal considerations in providing virtual care in Newfoundland and Labrador.

| Not Confident | Somewhat Confident | Confident | Very Confident |
| --- | --- | --- | --- |
| 1 | 2 | 3 | 4 |
