# Supplementary material for: Evaluation study of effect of virtual care education on healthcare providers’ knowledge, confidence, and satisfaction
Source: PeerJ. 2025 Nov 20;13:e20414. doi: 10.7717/peerj.20414 (PMC12640642; doi:10.7717/peerj.20414)
Supplement: Supplemental Information 6 [file peerj-13-20414-s006.pdf]

Explore

Notes

Output Created		21-JUL-2025 17:23:17
Comments		
Input	Data	/Users/meganclemens/Downloads/1C. Virtual Care - Analysis.sav
	Active Dataset	DataSet1
	Filter	<none>
	Weight	<none>
	Split File	<none>
	N of Rows in Working Data File	22
Missing Value Handling	Definition of Missing	User-defined missing values for dependent variables are treated as missing.
	Cases Used	Statistics are based on cases with no missing values for any dependent variable or factor used.
Syntax		EXAMINE VARIABLES=Knowledge_Total.1 Knowledge_Total.2<br>/PLOT BOXPLOT STEMLEAF HISTOGRAM NPPLOT<br>/COMPARE GROUPS<br>/STATISTICS DESCRIPTIVES<br>/CINTERVAL 95<br>/MISSING LISTWISE<br>/NOTOTAL.
Resources	Processor Time	00:00:01.65
	Elapsed Time	00:00:01.00

Case Processing Summary

	Cases					
	Valid		Missing		Total	
	N	Percent	N	Percent	N	Percent
Pre-test knowledge result	22	100.0%	0	0.0%	22	100.0%
Post-test knowledge result	22	100.0%	0	0.0%	22	100.0%

Descriptives

			Statistic	Std. Error
Pre-test knowledge result	Mean		5.18	.306
	95% Confidence Interval for Mean	Lower Bound	4.55	
		Upper Bound	5.82	
	5% Trimmed Mean		5.36	
	Median		6.00	
	Variance		2.061	
	Std. Deviation		1.435	
	Minimum		1	
	Maximum		6	
	Range		5	
	Interquartile Range		1	
	Skewness		-1.731	.491
	Kurtosis		2.230	.953
Post-test knowledge result	Mean		5.73	.097
	95% Confidence Interval for Mean	Lower Bound	5.53	
		Upper Bound	5.93	
	5% Trimmed Mean		5.75	
	Median		6.00	
	Variance		.208	
	Std. Deviation		.456	
	Minimum		5	
	Maximum		6	
	Range		1	
	Interquartile Range		1	
	Skewness		-1.097	.491
	Kurtosis		-.887	.953

Tests of Normality

	Kolmogorov-Smirnov[a]			Shapiro-Wilk		
	Statistic	df	Sig.	Statistic	df	Sig.
Pre-test knowledge result	.397	22	<.001	.640	22	<.001
Post-test knowledge result	.452	22	<.001	.561	22	<.001

a. Lilliefors Significance Correction

Pre-test knowledge result

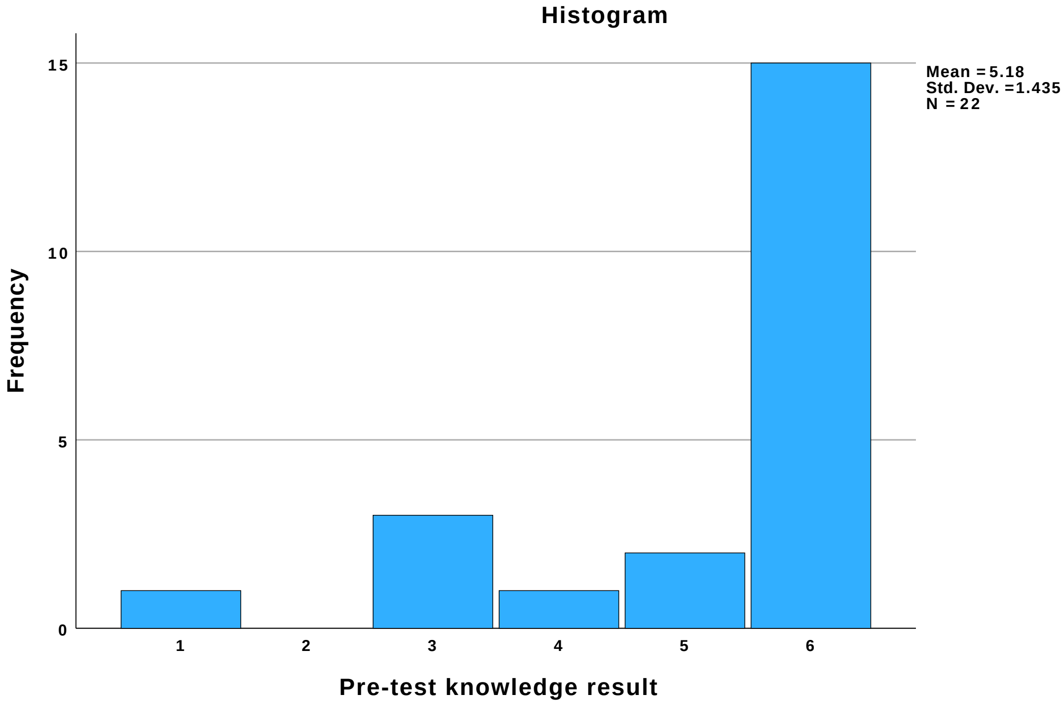

Post-test knowledge result

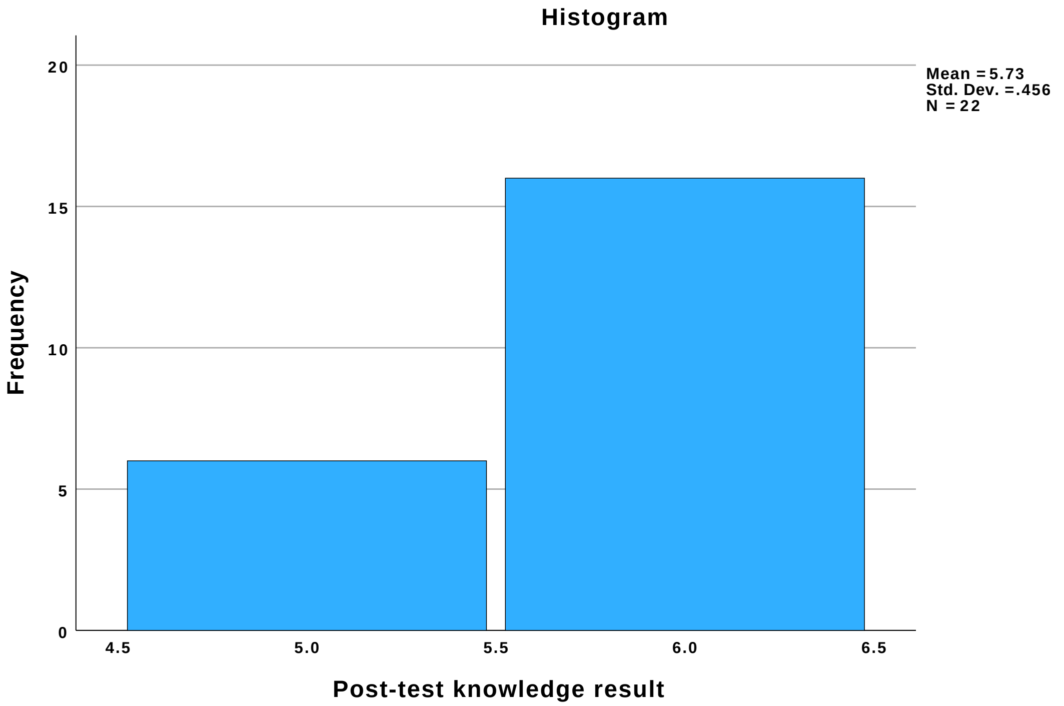

Notes

Output Created		21-JUL-2025 17:23:18
Comments		
Input	Data	/Users/meganclemens/Downloads/1C. Virtual Care - Analysis.sav
	Active Dataset	DataSet1
	Filter	<none>
	Weight	<none>
	Split File	<none>
	N of Rows in Working Data File	22
Missing Value Handling	Definition of Missing	User-defined missing values are treated as missing.
	Cases Used	Statistics are based on all cases with valid data.
Syntax		FREQUENCIES VARIABLES=Knowledge_Total.1 Knowledge_Total.2 /STATISTICS=MEAN /HISTOGRAM NORMAL ...
Resources	Processor Time	00:00:00.37
	Elapsed Time	00:00:00.00

Frequency Table

Pre-test knowledge result

		Frequency	Percent	Valid Percent	Cumulative Percent
Valid	1	1	4.5	4.5	4.5
	3	3	13.6	13.6	18.2
	4	1	4.5	4.5	22.7
	5	2	9.1	9.1	31.8
	6	15	68.2	68.2	100.0
	Total	22	100.0	100.0	

Post-test knowledge result

		Frequency	Percent	Valid Percent	Cumulative Percent
Valid	5	6	27.3	27.3	27.3
	6	16	72.7	72.7	100.0
	Total	22	100.0	100.0	

Notes

Output Created		21-JUL-2025 17:23:18
Comments		
Input	Data	/Users/meganclemens/Downloads/1C. Virtual Care - Analysis.sav
	Active Dataset	DataSet1
	Filter	<none>
	Weight	<none>
	Split File	<none>
	N of Rows in Working Data File	22
Syntax		NPTESTS /RELATED TEST (Knowledge_Total.1 Knowledge_Total.2) WILCOXON /MISSING SCOPE=ANALYSIS USERMISSING=EXCLUDE /CRITERIA ALPHA=0.05 CILEVEL=95.
Resources	Processor Time	00:00:00.50
	Elapsed Time	00:00:01.00

Related-Samples Wilcoxon Signed Rank Test Summary

Total N	22
Test Statistic	39.000
Standard Error	8.261
Standardized Test Statistic	1.997
Asymptotic Sig.(2-sided test)	.046

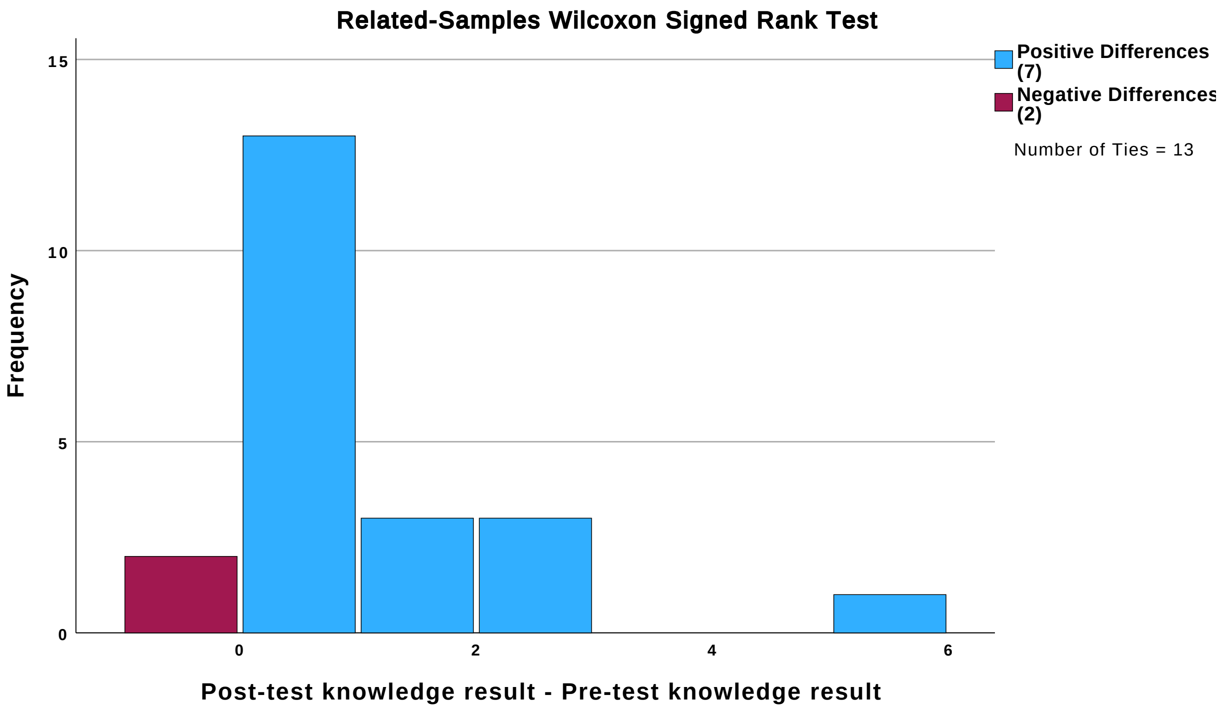

Frequencies

Notes

Output Created		21-JUL-2025 17:23:28
Comments		
Input	Data	/Users/meganclemens/Downloads/1C. Virtual Care - Analysis.sav
	Active Dataset	DataSet1
	Filter	<none>
	Weight	<none>
	Split File	<none>
	N of Rows in Working Data File	22
Missing Value Handling	Definition of Missing	User-defined missing values are treated as missing.
	Cases Used	Statistics are based on all cases with valid data.
Syntax		FREQUENCIES VARIABLES=Confidence_Pre Confidence_Post /STATISTICS=RANGE MEAN MEDIAN /ORDER=ANALYSIS.
Resources	Processor Time	00:00:00.01
	Elapsed Time	00:00:00.00

Statistics

		Confidence_Pre	Confidence_Post
N	Valid	21	20
	Missing	1	2
Mean		16.4762	21.3000
Median		17.0000	23.5000
Range		12.00	11.00

Frequency Table

Confidence_Pre

		Frequency	Percent	Valid Percent	Cumulative Percent
Valid	12.00	2	9.1	9.5	9.5
	13.00	2	9.1	9.5	19.0
	14.00	2	9.1	9.5	28.6
	15.00	1	4.5	4.8	33.3
	16.00	3	13.6	14.3	47.6
	17.00	5	22.7	23.8	71.4
	18.00	3	13.6	14.3	85.7
	19.00	1	4.5	4.8	90.5
	23.00	1	4.5	4.8	95.2
	24.00	1	4.5	4.8	100.0
	Total	21	95.5	100.0	
Missing	System	1	4.5		
Total		22	100.0		

Confidence_Post

		Frequency	Percent	Valid Percent	Cumulative Percent
Valid	13.00	1	4.5	5.0	5.0
	18.00	6	27.3	30.0	35.0
	19.00	1	4.5	5.0	40.0
	23.00	2	9.1	10.0	50.0
	24.00	10	45.5	50.0	100.0
	Total	20	90.9	100.0	
Missing	System	2	9.1		
Total		22	100.0		

Explore

Notes

Output Created		21-JUL-2025 17:23:33
Comments		
Input	Data	/Users/meganclemens/Downloads/1C. Virtual Care - Analysis.sav
	Active Dataset	DataSet1
	Filter	<none>
	Weight	<none>
	Split File	<none>
	N of Rows in Working Data File	22
Missing Value Handling	Definition of Missing	User-defined missing values for dependent variables are treated as missing.
	Cases Used	Statistics are based on cases with no missing values for any dependent variable or factor used.
Syntax		EXAMINE VARIABLES=Q7_Diff Q8_Diff Q9_Diff Q10_Diff Q11_Diff Q12_Diff<br>/PLOT BOXPLOT STEMLEAF HISTOGRAM NPPLOT<br>/COMPARE GROUPS<br>/STATISTICS DESCRIPTIVES<br>/CINTERVAL 95<br>/MISSING LISTWISE<br>/NOTOTAL.
Resources	Processor Time	00:00:03.62
	Elapsed Time	00:00:03.00

Case Processing Summary

	Cases					
	Valid		Missing		Total	
	N	Percent	N	Percent	N	Percent
Pre- to post-test confidence Q7	19	86.4%	3	13.6%	22	100.0%
Pre- to post-test confidence Q8	19	86.4%	3	13.6%	22	100.0%
Pre- to post-test confidence Q9	19	86.4%	3	13.6%	22	100.0%
Pre- to post-test confidence Q10	19	86.4%	3	13.6%	22	100.0%
Pre- to post-test confidence Q11	19	86.4%	3	13.6%	22	100.0%
Pre- to post-test confidence Q12	19	86.4%	3	13.6%	22	100.0%

Descriptives

			Statistic	Std. Error
Pre- to post-test confidence Q7	Mean		.79	.123
	95% Confidence Interval for Mean	Lower Bound	.53	
		Upper Bound	1.05	
	5% Trimmed Mean		.77	
	Median		1.00	
	Variance		.287	
	Std. Deviation		.535	
	Minimum		0	
	Maximum		2	
	Range		2	
	Interquartile Range		1	
	Skewness		-.229	.524
	Kurtosis		.316	1.014
Pre- to post-test confidence Q8	Mean		.74	.150
	95% Confidence Interval for Mean	Lower Bound	.42	
		Upper Bound	1.05	
	5% Trimmed Mean		.71	
	Median		1.00	
	Variance		.427	
	Std. Deviation		.653	
	Minimum		0	
	Maximum		2	
	Range		2	
	Interquartile Range		1	
	Skewness		.314	.524
	Kurtosis		-.506	1.014

Descriptives

			Statistic	Std. Error
Pre- to post-test confidence Q9	Mean		.68	.203
	95% Confidence Interval for Mean	Lower Bound	.26	
		Upper Bound	1.11	
	5% Trimmed Mean		.65	
	Median		1.00	
	Variance		.784	
	Std. Deviation		.885	
	Minimum		-1	
	Maximum		3	
	Range		4	
	Interquartile Range		1	
	Skewness		.713	.524
	Kurtosis		1.663	1.014
Pre- to post-test confidence Q10	Mean		.63	.157
	95% Confidence Interval for Mean	Lower Bound	.30	
		Upper Bound	.96	
	5% Trimmed Mean		.59	
	Median		1.00	
	Variance		.468	
	Std. Deviation		.684	
	Minimum		0	
	Maximum		2	
	Range		2	
	Interquartile Range		1	
	Skewness		.632	.524
	Kurtosis		-.527	1.014
Pre- to post-test confidence Q11	Mean		.74	.185
	95% Confidence Interval for Mean	Lower Bound	.35	
		Upper Bound	1.13	
	5% Trimmed Mean		.71	
	Median		1.00	
	Variance		.649	
	Std. Deviation		.806	
	Minimum		0	
	Maximum		2	
	Range		2	
	Interquartile Range		1	
	Skewness		.543	.524
	Kurtosis		-1.204	1.014
Pre- to post-test [illegible]	Mean		.89	.201

Descriptives

			Statistic	Std. Error
confidence Q12	95% Confidence Interval for Mean	Lower Bound	.47	
		Upper Bound	1.32	
	5% Trimmed Mean		.83	
	Median		1.00	
	Variance		.766	
	Std. Deviation		.875	
	Minimum		0	
	Maximum		3	
	Range		3	
	Interquartile Range		1	
	Skewness		.776	.524
	Kurtosis		.210	1.014

Tests of Normality

	Kolmogorov-Smirnov[a]			Shapiro-Wilk		
	Statistic	df	Sig.	Statistic	df	Sig.
Pre- to post-test confidence Q7	.390	19	<.001	.708	19	<.001
Pre- to post-test confidence Q8	.288	19	<.001	.784	19	<.001
Pre- to post-test confidence Q9	.255	19	.002	.857	19	.009
Pre- to post-test confidence Q10	.296	19	<.001	.770	19	<.001
Pre- to post-test confidence Q11	.293	19	<.001	.774	19	<.001
Pre- to post-test confidence Q12	.242	19	.005	.837	19	.004

a. Lilliefors Significance Correction

Pre- to post-test confidence Q7

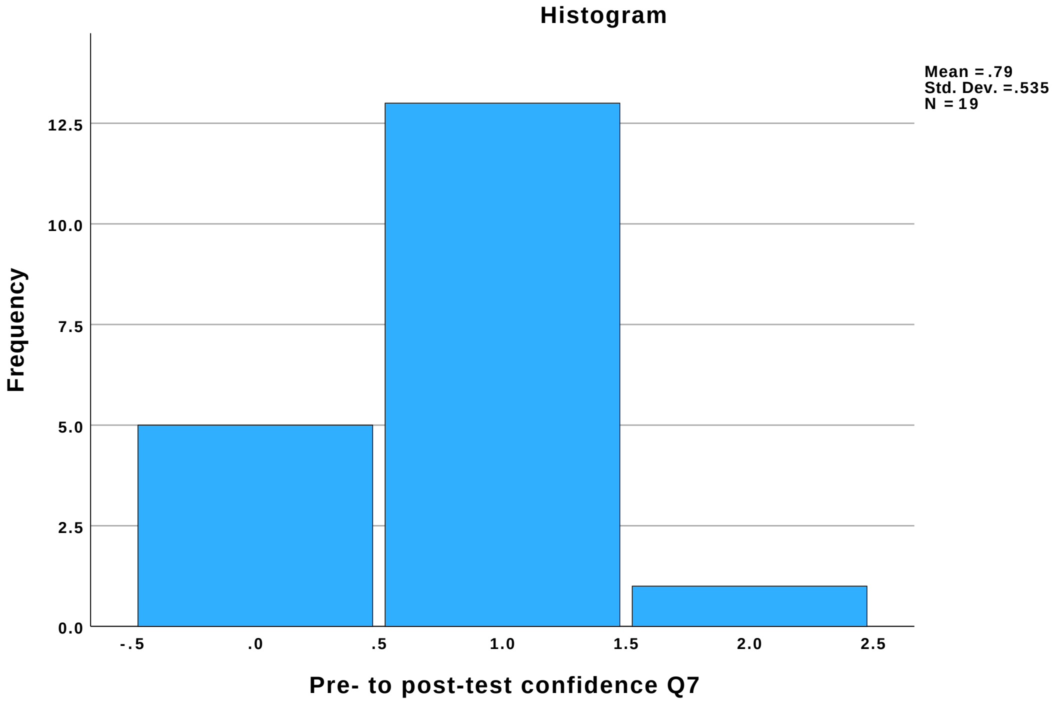

Pre- to post-test confidence Q8

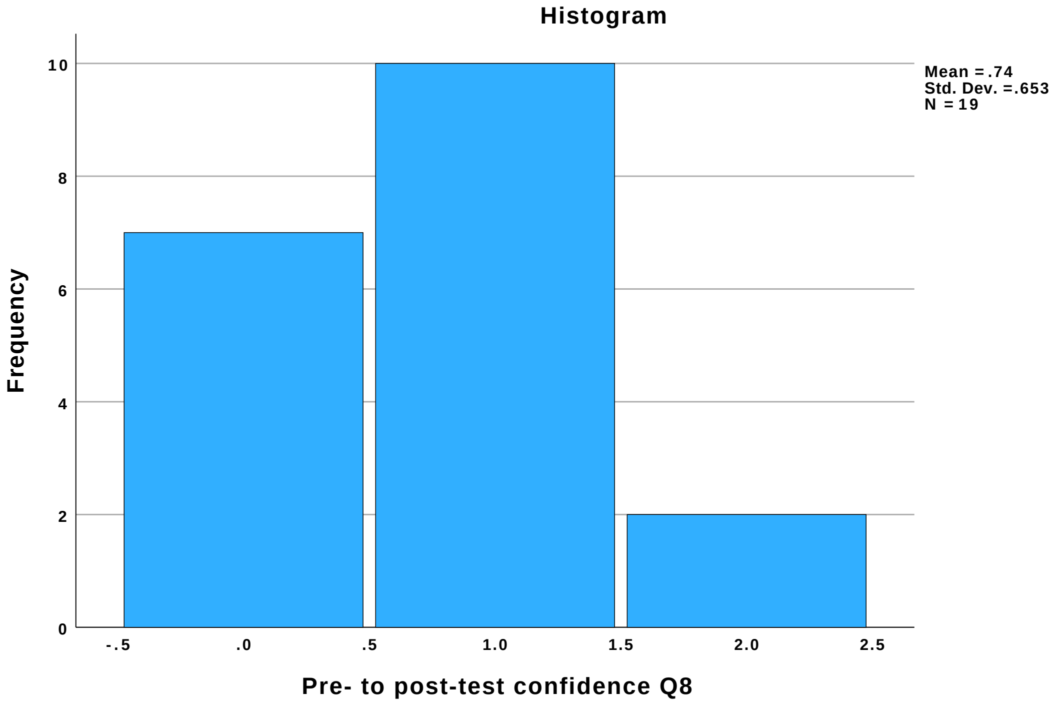

Pre- to post-test confidence Q9

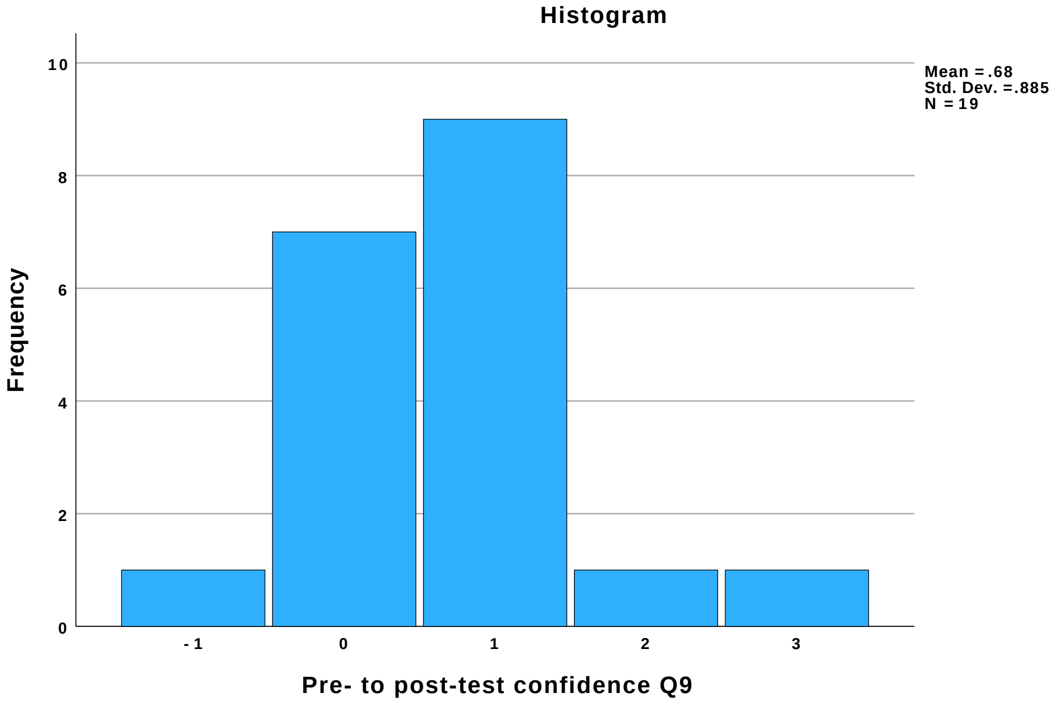

Pre- to post-test confidence Q10

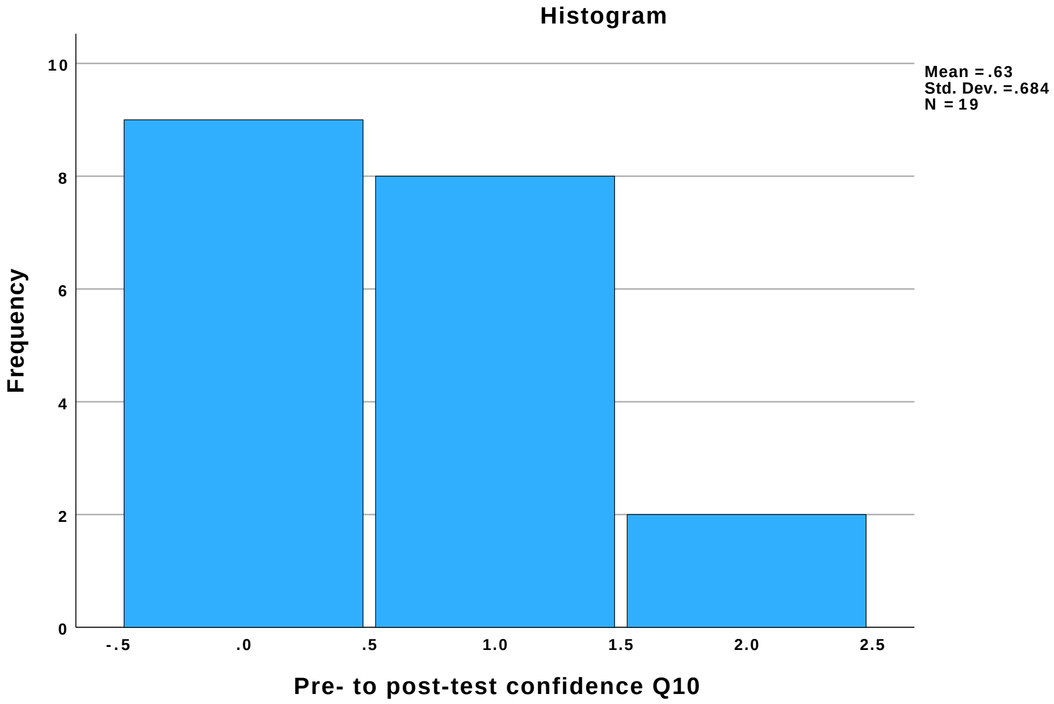

Pre- to post-test confidence Q11

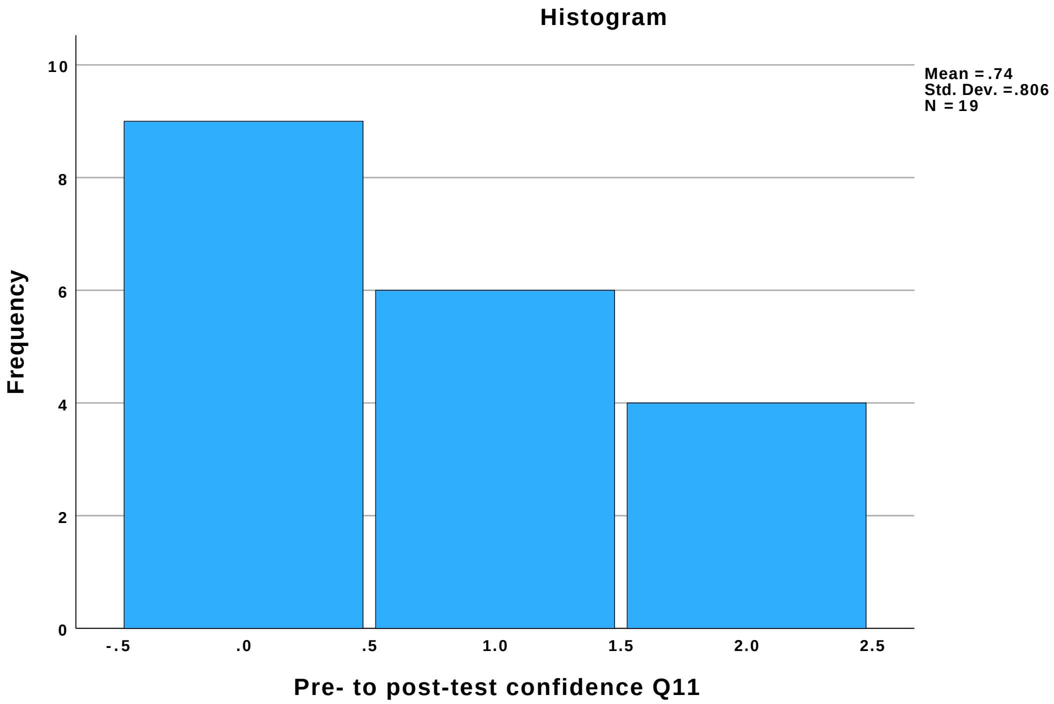

Pre- to post-test confidence Q12

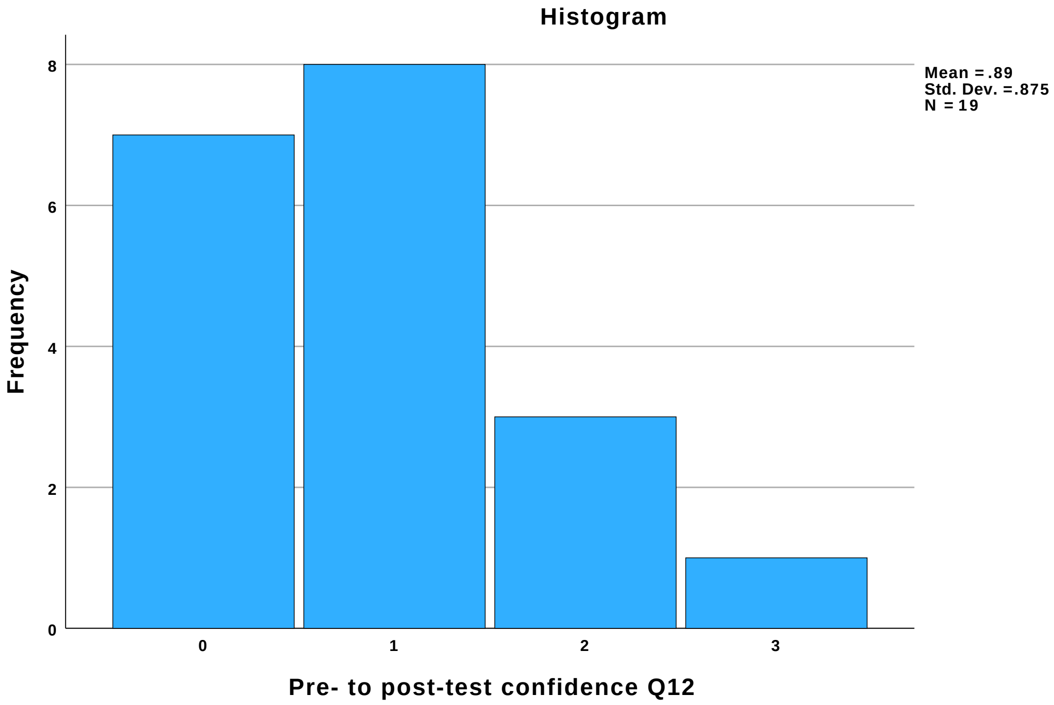

Notes

Output Created		21-JUL-2025 17:23:36
Comments		
Input	Data	/Users/meganclemens/Downloads/1C. Virtual Care - Analysis.sav
	Active Dataset	DataSet1
	Filter	<none>
	Weight	<none>
	Split File	<none>
	N of Rows in Working Data File	22
Syntax		NPTESTS /RELATED TEST(Q7.1 Q7.2) WILCOXON /MISSING SCOPE=ANALYSIS USERMISSING=EXCLUDE /CRITERIA ALPHA=0.05 CILEVEL=95.
Resources	Processor Time	00:00:00.66
	Elapsed Time	00:00:00.00

Related-Samples Wilcoxon Signed Rank Test

Pre-test confidence Q7. I can describe the benefits and key considerations of conducting virtual care appointments., Post-test confidence Q7. I can describe the benefits and key considerations of conducting virtual care appointments.

Related-Samples Wilcoxon Signed Rank Test Summary

Total N	21
Test Statistic	120.500
Standard Error	17.804
Standardized Test Statistic	2.949
Asymptotic Sig.(2-sided test)	.003

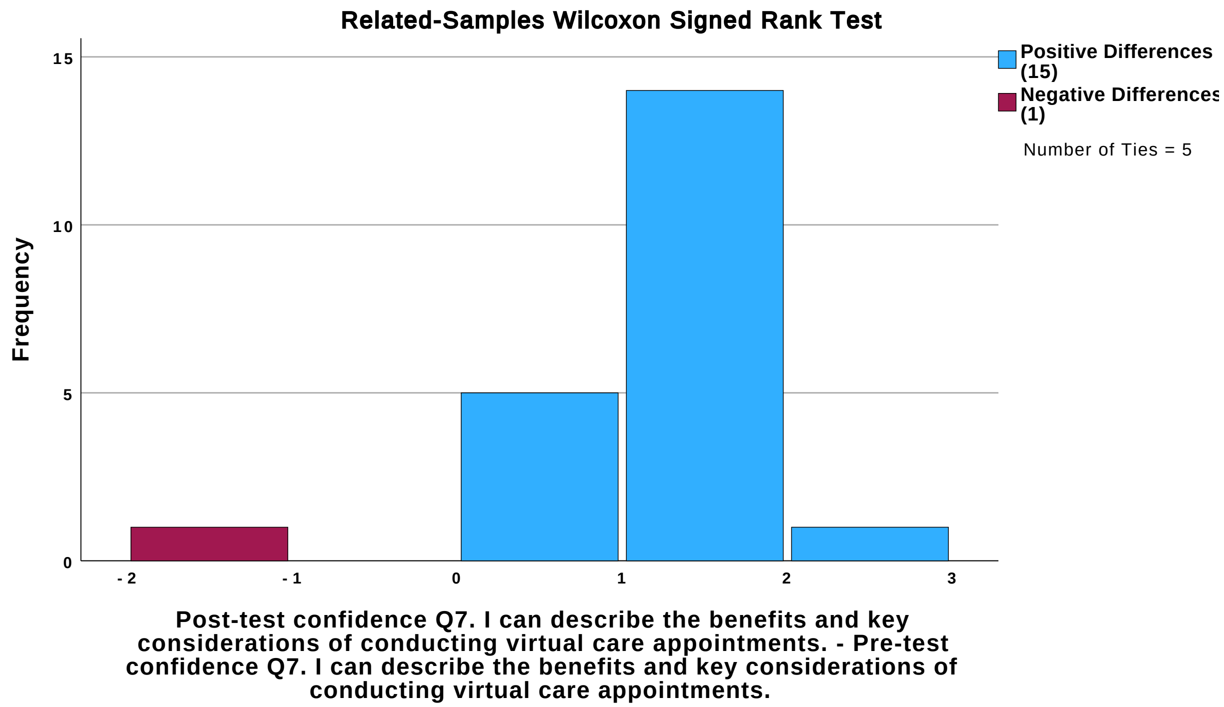

Notes

Output Created		21-JUL-2025 17:23:36
Comments		
Input	Data	/Users/meganclemens/Downloads/1C. Virtual Care - Analysis.sav
	Active Dataset	DataSet1
	Filter	<none>
	Weight	<none>
	Split File	<none>
	N of Rows in Working Data File	22
Syntax		NPTESTS /RELATED TEST(Q8.1 Q8.2) WILCOXON /MISSING SCOPE=ANALYSIS USERMISSING=EXCLUDE /CRITERIA ALPHA=0.05 CILEVEL=95.
Resources	Processor Time	00:00:00.43
	Elapsed Time	00:00:01.00

Related-Samples Wilcoxon Signed Rank Test

Pre-test confidence Q8. I can identify the technological requirements and setup required to conduct optimal virtual care., Post-test confidence Q8. I can identify the technological requirements and setup required to conduct optimal virtual care.

Related-Samples Wilcoxon Signed Rank Test Summary

Total N	20
Test Statistic	91.000
Standard Error	13.309
Standardized Test Statistic	3.419
Asymptotic Sig.(2-sided test)	<.001

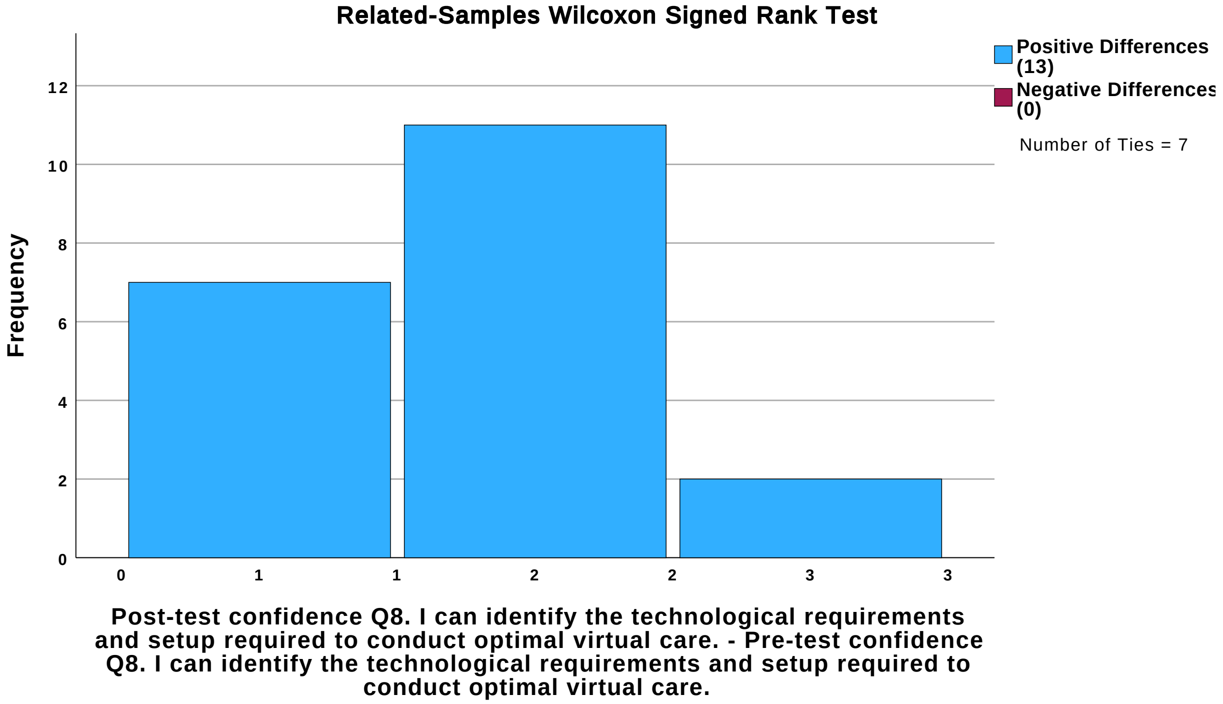

Notes

Output Created		21-JUL-2025 17:23:37
Comments		
Input	Data	/Users/meganclemens/Downloads/1C. Virtual Care - Analysis.sav
	Active Dataset	DataSet1
	Filter	<none>
	Weight	<none>
	Split File	<none>
	N of Rows in Working Data File	22
Syntax		NPTESTS /RELATED TEST(Q9.1 Q9.2) WILCOXON /MISSING SCOPE=ANALYSIS USERMISSING=EXCLUDE /CRITERIA ALPHA=0.05 CILEVEL=95.
Resources	Processor Time	00:00:00.48
	Elapsed Time	00:00:00.00

Related-Samples Wilcoxon Signed Rank Test

Pre-test confidence Q9. I recognize how to integrate virtual care delivery into my existing practice workflows., Post-test confidence Q9. I recognize how to integrate virtual care delivery into my existing practice workflows.

Related-Samples Wilcoxon Signed Rank Test Summary

Total N	20
Test Statistic	85.000
Standard Error	13.314
Standardized Test Statistic	2.967
Asymptotic Sig.(2-sided test)	.003

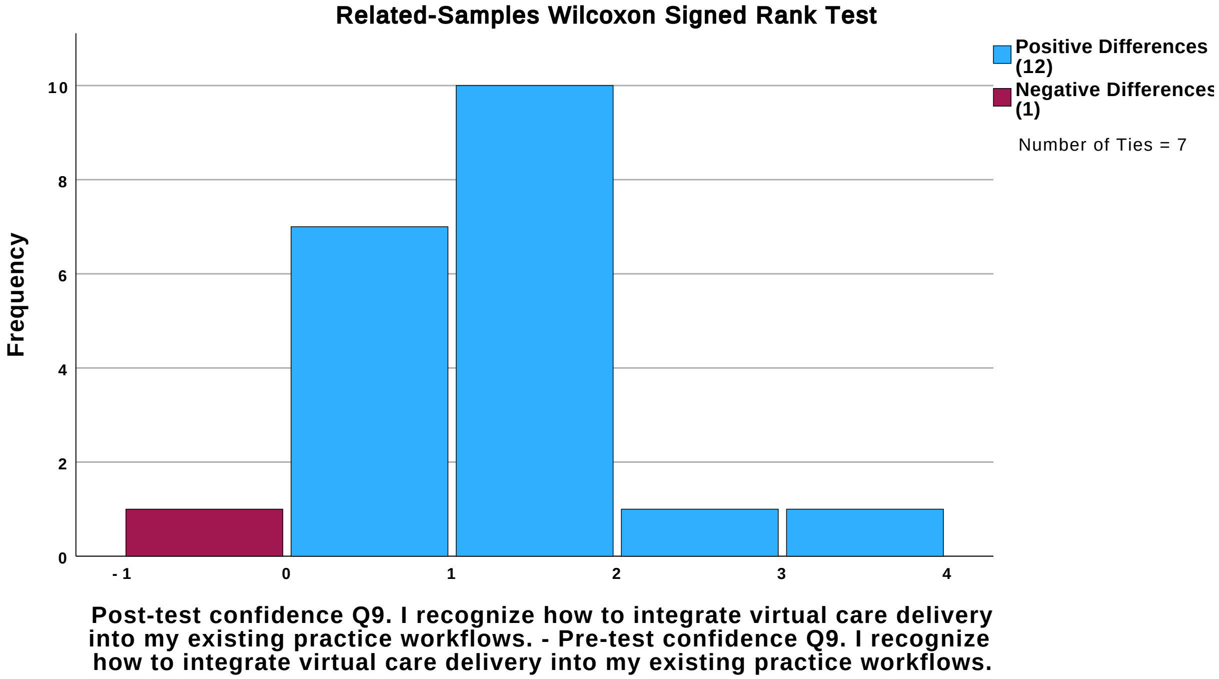

Notes

Output Created		21-JUL-2025 17:23:37
Comments		
Input	Data	/Users/meganclemens/Downloads/1C. Virtual Care - Analysis.sav
	Active Dataset	DataSet1
	Filter	<none>
	Weight	<none>
	Split File	<none>
	N of Rows in Working Data File	22
Syntax		NPTESTS /RELATED TEST(Q10.1 Q10.2) WILCOXON /MISSING SCOPE=ANALYSIS USERMISSING=EXCLUDE /CRITERIA ALPHA=0.05 CILEVEL=95.
Resources	Processor Time	00:00:00.47
	Elapsed Time	00:00:01.00

Related-Samples Wilcoxon Signed Rank Test

Pre-test confidence Q10. I can discuss the clinical implications for delivering optimal virtual care encounters., Post-test confidence Q10. I can discuss the clinical implications for delivering optimal virtual care encounters.

Related-Samples Wilcoxon Signed Rank Test Summary

Total N	20
Test Statistic	66.000
Standard Error	10.553
Standardized Test Statistic	3.127
Asymptotic Sig.(2-sided test)	.002

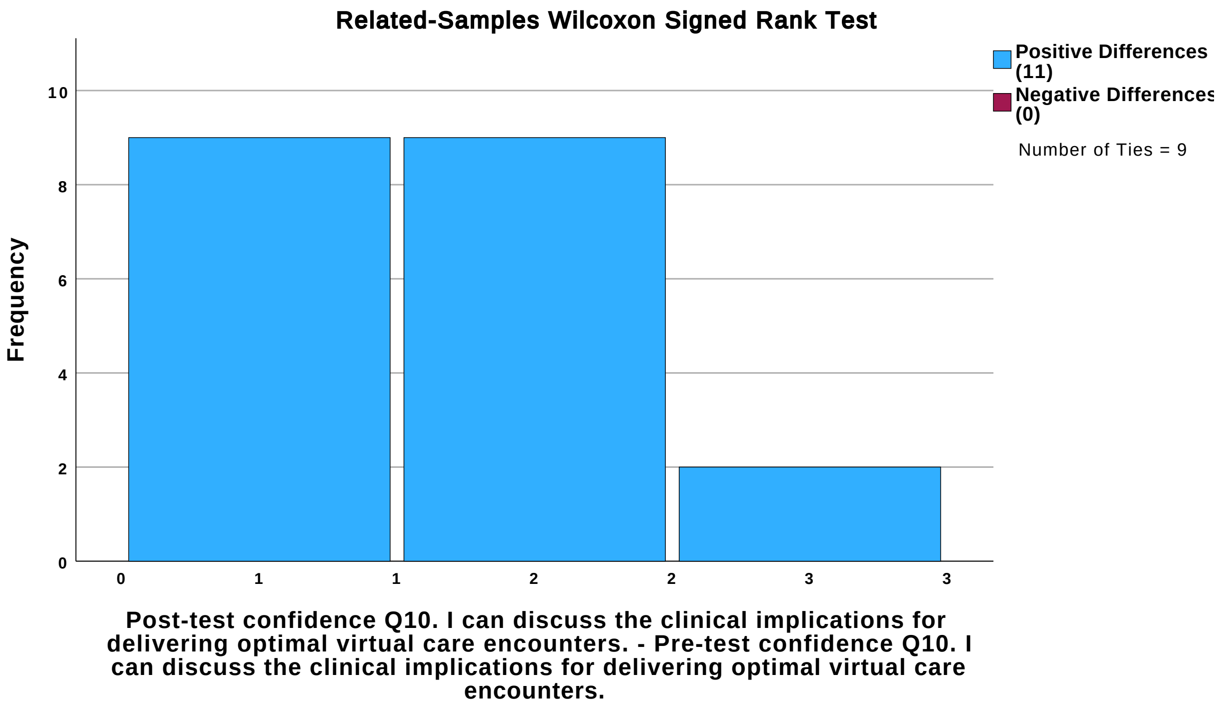

Notes

Output Created		21-JUL-2025 17:23:38
Comments		
Input	Data	/Users/meganclemens/Downloads/1C. Virtual Care - Analysis.sav
	Active Dataset	DataSet1
	Filter	<none>
	Weight	<none>
	Split File	<none>
	N of Rows in Working Data File	22
Syntax		NPTESTS<br>/RELATED TEST(Q11.1 Q11.2) WILCOXON<br>/MISSING SCOPE=ANALYSIS USERMISSING=EXCLUDE<br>/CRITERIA ALPHA=0.05 CILEVEL=95.
Resources	Processor Time	00:00:00.54
	Elapsed Time	00:00:00.00

Related-Samples Wilcoxon Signed Rank Test

Pre-test confidence Q11. I can explain how to prepare patients for virtual care sessions., Post-test confidence Q11. I can explain how to prepare patients for virtual care sessions.

Related-Samples Wilcoxon Signed Rank Test Summary

Total N	20
Test Statistic	66.000
Standard Error	10.874
Standardized Test Statistic	3.035
Asymptotic Sig.(2-sided test)	.002

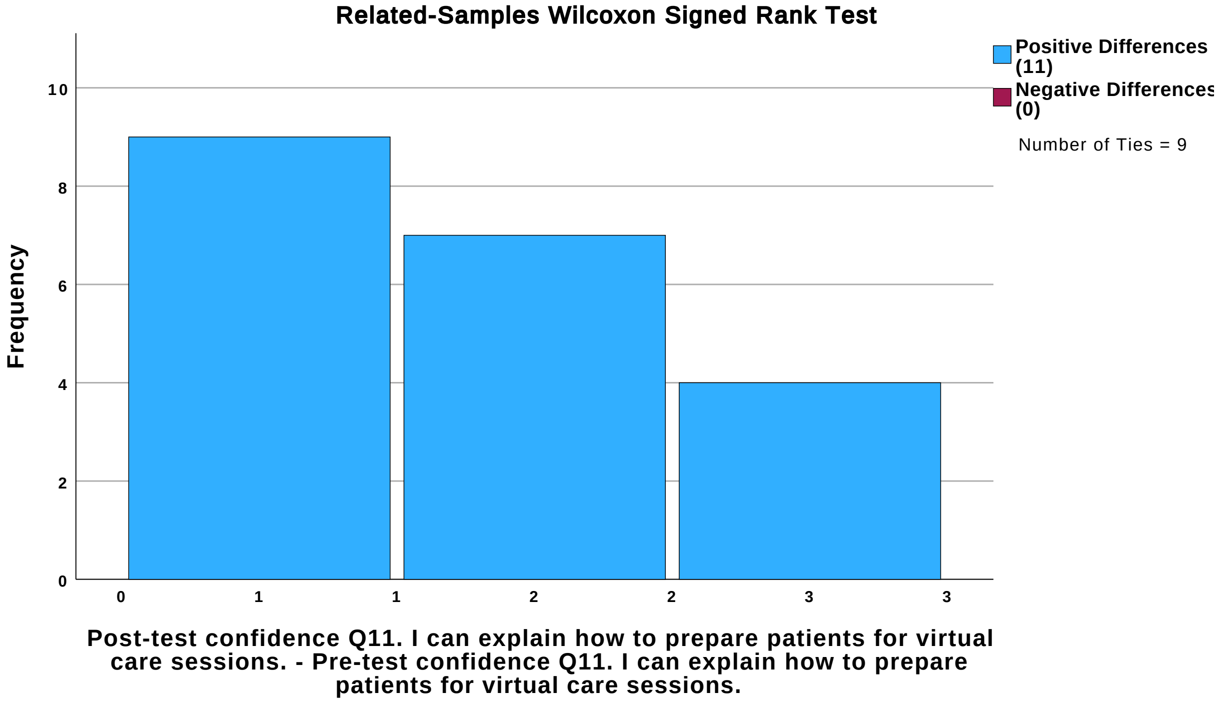

Notes

Output Created		21-JUL-2025 17:23:38
Comments		
Input	Data	/Users/meganclemens/Downloads/1C. Virtual Care - Analysis.sav
	Active Dataset	DataSet1
	Filter	<none>
	Weight	<none>
	Split File	<none>
	N of Rows in Working Data File	22
Syntax		NPTESTS /RELATED TEST(Q12.1 Q12.2) WILCOXON /MISSING SCOPE=ANALYSIS USERMISSING=EXCLUDE /CRITERIA ALPHA=0.05 CILEVEL=95.
Resources	Processor Time	00:00:00.50
	Elapsed Time	00:00:00.00

Related-Samples Wilcoxon Signed Rank Test

Pre-test confidence Q12. I can summarize the key regulatory and legal considerations in providing virtual care in Newfoundland and Labrador., Post-test confidence Q12. I can summarize the key regulatory and legal considerations in providing virtual care in Newfoundland and Labrador.

Related-Samples Wilcoxon Signed Rank Test Summary

Total N	19
Test Statistic	78.000
Standard Error	12.309
Standardized Test Statistic	3.169
Asymptotic Sig.(2-sided test)	.002

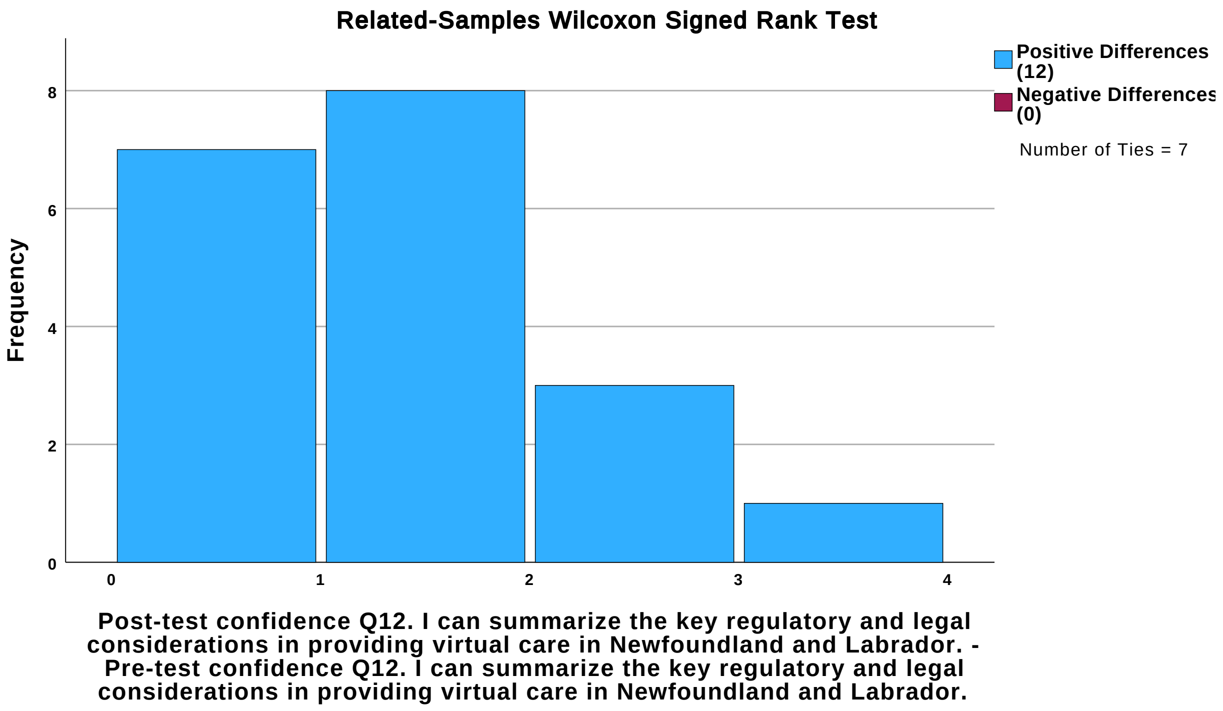

Your license will expire in 9 days.
