# Supplementary material for: Evaluation study of effect of virtual care education on healthcare providers’ knowledge, confidence, and satisfaction
Source: PeerJ. 2025 Nov 20;13:e20414. doi: 10.7717/peerj.20414 (PMC12640642; doi:10.7717/peerj.20414)
Supplement: Supplemental Information 9 [file peerj-13-20414-s009.docx]

**File Information**

| **Notes** | | |
| --- | --- | --- |
| Output Created | | 17-OCT-2024 12:32:47 |
| Comments | |  |
| Input | Data | C:\Users\geraldw\Desktop\Effective Virtual Care_October 17, 2024_07.02.sav |
|  | Active Dataset | DataSet1 |
|  | Filter | <none> |
|  | Weight | <none> |
|  | Split File | <none> |
| Syntax | | DISPLAY DICTIONARY. |
| Resources | Processor Time | 00:00:00.00 |
|  | Elapsed Time | 00:00:00.01 |

[DataSet1] C:\Users\geraldw\Desktop\Effective Virtual Care_October 17, 2024_07.02.sav

| **Variable Information** | | | | | | | | |
| --- | --- | --- | --- | --- | --- | --- | --- | --- |
| Variable | Position | Label | Measurement Level | Role | Column Width | Alignment | Print Format | Write Format |
| StartDate | 1 | Start Date | Scale | Input | 23 | Right | DATETIME20 | DATETIME20 |
| EndDate | 2 | End Date | Scale | Input | 5 | Right | DATETIME20 | DATETIME20 |
| Status | 3 | Response Type | Scale | Input | 5 | Right | F40 | F40 |
| IPAddress | 4 | IP Address | Nominal | Input | 15 | Left | A2000 | A2000 |
| Progress | 5 | Progress | Scale | Input | 5 | Right | F40.2 | F40.2 |
| Duration__in_seconds_ | 6 | Duration (in seconds) | Scale | Input | 5 | Right | F40.2 | F40.2 |
| Finished | 7 | Finished | Scale | Input | 5 | Right | F40 | F40 |
| RecordedDate | 8 | Recorded Date | Scale | Input | 5 | Right | DATETIME20 | DATETIME20 |
| ResponseId | 9 | Response ID | Nominal | Input | 15 | Left | A50 | A50 |
| RecipientLastName | 10 | Recipient Last Name | Nominal | Input | 15 | Left | A2000 | A2000 |
| RecipientFirstName | 11 | Recipient First Name | Nominal | Input | 15 | Left | A2000 | A2000 |
| RecipientEmail | 12 | Recipient Email | Nominal | Input | 15 | Left | A2000 | A2000 |
| ExternalReference | 13 | External Data Reference | Nominal | Input | 15 | Left | A2000 | A2000 |
| LocationLatitude | 14 | Location Latitude | Nominal | Input | 15 | Left | A2000 | A2000 |
| LocationLongitude | 15 | Location Longitude | Nominal | Input | 15 | Left | A2000 | A2000 |
| DistributionChannel | 16 | Distribution Channel | Nominal | Input | 15 | Left | A2000 | A2000 |
| UserLanguage | 17 | User Language | Nominal | Input | 15 | Left | A2000 | A2000 |
| Q2_1 | 18 | 1. Course Content: - Addressed my learning needs. | Scale | Input | 5 | Right | F40 | F40 |
| Q2_2 | 19 | 1. Course Content: - Enhanced my knowledge. | Scale | Input | 5 | Right | F40 | F40 |
| Q2_3 | 20 | 1. Course Content: - Met the stated objectives. | Scale | Input | 5 | Right | F40 | F40 |
| Q2_4 | 21 | 1. Course Content: - Was relevant to my practice. | Scale | Input | 5 | Right | F40 | F40 |
| Q2_5 | 22 | 1. Course Content: - Disclosed potential conflicts of interest. | Scale | Input | 5 | Right | F40 | F40 |
| Q2_6 | 23 | 1. Course Content: - Disclosed commercial support. | Scale | Input | 5 | Right | F40 | F40 |
| Q2_7 | 24 | 1. Course Content: - Was in keeping with the mission of the Office of Professional and Educational Development.* | Scale | Input | 5 | Right | F40 | F40 |
| Q4_1 | 25 | 2. Please indicate which CanMEDS-FM roles* you felt were addressed during this educational activity (check all that apply): Family Medicine Expert / Medical Expert | Scale | Input | 5 | Right | F40 | F40 |
| Q4_2 | 26 | 2. Please indicate which CanMEDS-FM roles* you felt were addressed during this educational activity (check all that apply): Communicator | Scale | Input | 5 | Right | F40 | F40 |
| Q4_3 | 27 | 2. Please indicate which CanMEDS-FM roles* you felt were addressed during this educational activity (check all that apply): Collaborator | Scale | Input | 5 | Right | F40 | F40 |
| Q4_4 | 28 | 2. Please indicate which CanMEDS-FM roles* you felt were addressed during this educational activity (check all that apply): Manager/Leader | Scale | Input | 5 | Right | F40 | F40 |
| Q4_5 | 29 | 2. Please indicate which CanMEDS-FM roles* you felt were addressed during this educational activity (check all that apply): Health Advocate | Scale | Input | 5 | Right | F40 | F40 |
| Q4_6 | 30 | 2. Please indicate which CanMEDS-FM roles* you felt were addressed during this educational activity (check all that apply): Scholar | Scale | Input | 5 | Right | F40 | F40 |
| Q4_7 | 31 | 2. Please indicate which CanMEDS-FM roles* you felt were addressed during this educational activity (check all that apply): Professional | Scale | Input | 5 | Right | F40 | F40 |
| Q5 | 32 | 3. Did you perceive any degree of bias in any part of the course? | Scale | Input | 5 | Right | F40 | F40 |
| Q6 | 33 | If yes, please explain: | Nominal | Input | 15 | Left | A2000 | A2000 |
| Q26_1 | 34 | 4. Course Navigation/Technical Support: - Instruction on use of and access to the course was helpful. | Scale | Input | 5 | Right | F40 | F40 |
| Q26_2 | 35 | 4. Course Navigation/Technical Support: - The pages were well-organized. | Scale | Input | 5 | Right | F40 | F40 |
| Q26_3 | 36 | 4. Course Navigation/Technical Support: - The pages were easy to navigate. | Scale | Input | 5 | Right | F40 | F40 |
| Q26_4 | 37 | 4. Course Navigation/Technical Support: - I received adequate help with technical problems. | Scale | Input | 5 | Right | F40 | F40 |
| Q27_1 | 38 | 5. Interactive Components: - Participation in the discussion activities enhanced my understanding of the content. | Scale | Input | 5 | Right | F40 | F40 |
| Q27_2 | 39 | 5. Interactive Components: - Being provided with the opportunity to communicate with peers was helpful. | Scale | Input | 5 | Right | F40 | F40 |
| Q27_3 | 40 | 5. Interactive Components: - The discussion component was easy to use. | Scale | Input | 5 | Right | F40 | F40 |
| Q27_4 | 41 | 5. Interactive Components: - The "Ask the Expert" option addressed my learning needs. | Scale | Input | 5 | Right | F40 | F40 |
| Q9 | 42 | 6. What did you like about this course? | Nominal | Input | 15 | Left | A2000 | A2000 |
| Q10 | 43 | 7. How could we have improved this course? | Nominal | Input | 15 | Left | A2000 | A2000 |
| Q28 | 44 | 8. Suggestions for future continuing health education topics: | Nominal | Input | 15 | Left | A2000 | A2000 |
| Q29 | 45 | 9. Suggestions for non-clinical topics (i.e. related to communication, advocacy, leadership, etc.): | Nominal | Input | 15 | Left | A2000 | A2000 |
| Q16 | 46 | 10. Profession: - Selected Choice | Scale | Input | 5 | Right | F40 | F40 |
| Q16_9_TEXT | 47 | 10. Profession: - Other (please specify): - Text | Nominal | Input | 15 | Left | A2000 | A2000 |
| Q17_1 | 48 | 11. Type of Practice (check all that apply): - Selected Choice Solo | Scale | Input | 5 | Right | F40 | F40 |
| Q17_2 | 49 | 11. Type of Practice (check all that apply): - Selected Choice Group | Scale | Input | 5 | Right | F40 | F40 |
| Q17_3 | 50 | 11. Type of Practice (check all that apply): - Selected Choice Hospital-Based/Institution | Scale | Input | 5 | Right | F40 | F40 |
| Q17_4 | 51 | 11. Type of Practice (check all that apply): - Selected Choice Other (please specify): | Scale | Input | 5 | Right | F40 | F40 |
| Q17_4_TEXT | 52 | 11. Type of Practice (check all that apply): - Other (please specify): - Text | Nominal | Input | 15 | Left | A2000 | A2000 |
| Q18 | 53 | 12. Years of Experience: | Scale | Input | 5 | Right | F40 | F40 |
| Q20 | 54 | 13. Population of Community of Practice: | Scale | Input | 5 | Right | F40 | F40 |
| Q21 | 55 | 14. Gender: - Selected Choice | Scale | Input | 5 | Right | F40 | F40 |
| Q21_4_TEXT | 56 | 14. Gender: - Another Gender Identity (optional to specify): - Text | Nominal | Input | 15 | Left | A2000 | A2000 |
| Q19 | 57 | 15. Practice Setting: - Selected Choice | Scale | Input | 5 | Right | F40 | F40 |
| Q19_7_TEXT | 58 | 15. Practice Setting: - Other (please specify): - Text | Nominal | Input | 15 | Left | A2000 | A2000 |
| Q23 | 59 | 16. Please describe how you will change your practice as a result of participating in this course: | Nominal | Input | 15 | Left | A2000 | A2000 |
| Q30 | 60 | 17. Major population health needs in your practice area: | Nominal | Input | 15 | Left | A2000 | A2000 |
| Q24_1 | 61 | 18. How did you hear about this course? - Selected Choice My Employer | Scale | Input | 5 | Right | F40 | F40 |
| Q24_2 | 62 | 18. How did you hear about this course? - Selected Choice E-mail | Scale | Input | 5 | Right | F40 | F40 |
| Q24_3 | 63 | 18. How did you hear about this course? - Selected Choice Newsletter | Scale | Input | 5 | Right | F40 | F40 |
| Q24_5 | 64 | 18. How did you hear about this course? - Selected Choice Website | Scale | Input | 5 | Right | F40 | F40 |
| Q24_6 | 65 | 18. How did you hear about this course? - Selected Choice Colleague | Scale | Input | 5 | Right | F40 | F40 |
| Q24_4 | 66 | 18. How did you hear about this course? - Selected Choice Professional Association | Scale | Input | 5 | Right | F40 | F40 |
| Q24_7 | 67 | 18. How did you hear about this course? - Selected Choice Other (please specify): | Scale | Input | 5 | Right | F40 | F40 |
| Variables in the working file | | | | | | | | |

| **Variable Values** | | |
| --- | --- | --- |
| Value | | Label |
| Status | 0 | IP Address |
|  | 1 | Survey Preview |
|  | 2 | Survey Test |
|  | 4 | Imported |
|  | 8 | Spam |
|  | 9 | Survey Preview Spam |
|  | 12 | Imported Spam |
|  | 16 | Offline |
|  | 17 | Offline Survey Preview |
|  | 32 | EX |
|  | 40 | EX Spam |
|  | 48 | EX Offline |
| Finished | 0 | False |
|  | 1 | True |
| Q2_1 | 1 | Strongly Disagree |
|  | 2 | Disagree |
|  | 3 | Neutral |
|  | 4 | Agree |
|  | 5 | Strongly Agree |
|  | 6 | Not Applicable |
| Q2_2 | 1 | Strongly Disagree |
|  | 2 | Disagree |
|  | 3 | Neutral |
|  | 4 | Agree |
|  | 5 | Strongly Agree |
|  | 6 | Not Applicable |
| Q2_3 | 1 | Strongly Disagree |
|  | 2 | Disagree |
|  | 3 | Neutral |
|  | 4 | Agree |
|  | 5 | Strongly Agree |
|  | 6 | Not Applicable |
| Q2_4 | 1 | Strongly Disagree |
|  | 2 | Disagree |
|  | 3 | Neutral |
|  | 4 | Agree |
|  | 5 | Strongly Agree |
|  | 6 | Not Applicable |
| Q2_5 | 1 | Strongly Disagree |
|  | 2 | Disagree |
|  | 3 | Neutral |
|  | 4 | Agree |
|  | 5 | Strongly Agree |
|  | 6 | Not Applicable |
| Q2_6 | 1 | Strongly Disagree |
|  | 2 | Disagree |
|  | 3 | Neutral |
|  | 4 | Agree |
|  | 5 | Strongly Agree |
|  | 6 | Not Applicable |
| Q2_7 | 1 | Strongly Disagree |
|  | 2 | Disagree |
|  | 3 | Neutral |
|  | 4 | Agree |
|  | 5 | Strongly Agree |
|  | 6 | Not Applicable |
| Q4_1 | 1 | Family Medicine Expert / Medical Expert |
| Q4_2 | 1 | Communicator |
| Q4_3 | 1 | Collaborator |
| Q4_4 | 1 | Manager/Leader |
| Q4_5 | 1 | Health Advocate |
| Q4_6 | 1 | Scholar |
| Q4_7 | 1 | Professional |
| Q5 | 1 | Yes |
|  | 2 | No |
| Q26_1 | 1 | Strongly Disagree |
|  | 2 | Disagree |
|  | 3 | Neutral |
|  | 4 | Agree |
|  | 5 | Strongly Agree |
|  | 6 | Not Applicable |
| Q26_2 | 1 | Strongly Disagree |
|  | 2 | Disagree |
|  | 3 | Neutral |
|  | 4 | Agree |
|  | 5 | Strongly Agree |
|  | 6 | Not Applicable |
| Q26_3 | 1 | Strongly Disagree |
|  | 2 | Disagree |
|  | 3 | Neutral |
|  | 4 | Agree |
|  | 5 | Strongly Agree |
|  | 6 | Not Applicable |
| Q26_4 | 1 | Strongly Disagree |
|  | 2 | Disagree |
|  | 3 | Neutral |
|  | 4 | Agree |
|  | 5 | Strongly Agree |
|  | 6 | Not Applicable |
| Q27_1 | 1 | Strongly Disagree |
|  | 2 | Disagree |
|  | 3 | Neutral |
|  | 4 | Agree |
|  | 5 | Strongly Agree |
|  | 6 | Not Applicable |
| Q27_2 | 1 | Strongly Disagree |
|  | 2 | Disagree |
|  | 3 | Neutral |
|  | 4 | Agree |
|  | 5 | Strongly Agree |
|  | 6 | Not Applicable |
| Q27_3 | 1 | Strongly Disagree |
|  | 2 | Disagree |
|  | 3 | Neutral |
|  | 4 | Agree |
|  | 5 | Strongly Agree |
|  | 6 | Not Applicable |
| Q27_4 | 1 | Strongly Disagree |
|  | 2 | Disagree |
|  | 3 | Neutral |
|  | 4 | Agree |
|  | 5 | Strongly Agree |
|  | 6 | Not Applicable |
| Q16 | 1 | General Practitioner / Family Physician |
|  | 2 | Specialist |
|  | 3 | Resident |
|  | 4 | Medical Student |
|  | 5 | Nurse Practitioner |
|  | 6 | Registered Nurse |
|  | 7 | Pharmacist |
|  | 8 | Social Worker |
|  | 9 | Other (please specify): |
|  | 10 | Physiotherapist |
|  | 11 | Occupational Therapist |
| Q17_1 | 1 | Solo |
| Q17_2 | 1 | Group |
| Q17_3 | 1 | Hospital-Based/Institution |
| Q17_4 | 1 | Other (please specify): |
| Q18 | 1 | 0-5 years |
|  | 2 | 6-10 years |
|  | 3 | 11-15 years |
|  | 4 | 16-20 years |
|  | 5 | >20 years |
| Q20 | 1 | <2,000 |
|  | 2 | 2,000-9,999 |
|  | 3 | 10,000-20,000 |
|  | 4 | >20,000 |
| Q21 | 1 | Male |
|  | 2 | Female |
|  | 3 | I do not wish to answer |
|  | 4 | Another Gender Identity (optional to specify): |
| Q19 | 1 | Hospital |
|  | 2 | Walk-in Clinic |
|  | 3 | Specialty Clinic |
|  | 4 | Community Health Centre |
|  | 5 | Family Health Team |
|  | 6 | Private Practice |
|  | 7 | Other (please specify): |
| Q24_1 | 1 | My Employer |
| Q24_2 | 1 | E-mail |
| Q24_3 | 1 | Newsletter |
| Q24_5 | 1 | Website |
| Q24_6 | 1 | Colleague |
| Q24_4 | 1 | Professional Association |
| Q24_7 | 1 | Other (please specify): |
